# Effect of PCSK9 Inhibitor on Blood Lipid Levels in Patients with High and Very-High CVD Risk: A Systematic Review and Meta-Analysis

**DOI:** 10.1155/2022/8729003

**Published:** 2022-04-26

**Authors:** Yue Zhang, Yanrong Suo, Lin Yang, Xiaolu Zhang, Qun Yu, Miao Zeng, Wenlan Zhang, Xijuan Jiang, Yijing Wang

**Affiliations:** ^1^School of Integrative Medicine, Tianjin University of Traditional Chinese Medicine, Tianjin, China; ^2^Traditional Chinese Medicine Department, Ganzhou People's Hospital, Ganzhou, China; ^3^School of Nursing, Tianjin University of Traditional Chinese Medicine, Tianjin, China

## Abstract

**Objectives:**

We aimed to investigate the effects of proprotein convertase subtilisin/kexin type 9 (PCSK9) inhibitor on blood lipid levels in patients with high and very-high cardiovascular risk.

**Design:**

14 trials (*n* = 52,586 patients) comparing treatment with or without PCSK9 inhibitors were retrieved from PubMed and Embase updated to 1st Jun 2021. The data quality of included studies was assessed by two independent researchers using the Cochrane systematic review method. All-cause mortality, cardiovascular mortality, and changes in serum low-density lipoprotein cholesterol (LDL-C), total cholesterol (TC), triglyceride (TG), apolipoprotein B (ApoB), lipoprotein (a) (LP (a)), non-high-density lipoprotein cholesterol (non-HDL-C), high-density lipoprotein cholesterol (HDL-C), and apolipoprotein A1 (ApoA1) from baseline were analyzed using Rev Man 5.1.0 software.

**Results:**

Compared with treatments without PCSK9 inhibitor, addition of PCSK9 inhibitors (evolocumab and alirocumab) had obvious decreasing effects on the levels of LDL-C [MD = −46.86, 95% CI (−54.99 to −38.72), *P* < 0.00001], TC [MD = −31.92, 95% CI (−39.47 to −24.38), *P* < 0.00001], TG [MD = −8.13, 95% CI (−10.48 to −5.79), *P* < 0.00001], LP(a) [MD = −26.69, 95% CI (-27.93 to −25.44), *P* < 0.00001], non-HDL-C [MD = −42.86, 95% CI (−45.81 to −39.92), *P* < 0.00001], and ApoB [MD = −38.44, 95% CI (−42.23 to -34.65), *P* < 0.00001] in high CVD risk patients. Conversely, changes of HDL-C [MD = 6.27, CI (5.17 to 7.36), *P* < 0.00001] and ApoA1 [MD = 4.33, 95% CI (3.53 to 5.13), *P* < 0.00001] from baseline were significantly more in high cardiovascular disease risk patients who received PCSK9 inhibitors treatment.

**Conclusion:**

Addition of PCSK9 inhibitors to standard therapy resulted in definite improvement in blood lipid levels compared with therapies that did not include them.

## 1. Introduction

Cardiovascular disease (CVD) remains the highest cause of morbidity and mortality worldwide [1]. A significant portion of CVD can be attributed to underlying coronary artery disease (CAD) with atherosclerosis, termed as atherosclerotic cardiovascular disease (ASCVD) [2, 3]. The important risk factor of ASCVD is dyslipidemia that includes hypertriglyceridemia, hypercholesterolemia, higher low-density lipoproteinemia (LDL), and mixed hyperlipidemia [4]. Meanwhile, non-high-density lipoprotein cholesterol (non-HDL-C) [5], such as lipoprotein (a) (Lp (a)) and very low-density lipoprotein cholesterol (VLDL-C), is also involved in the progression of ASCVD [6, 7]. Thus, management of dyslipidemia is a key component of primary and secondary risk-reduction strategies.

The previous guidelines classified CVD risk into four categories, low, moderate, high, and very-high risk, based on systematic coronary risk estimation (SCORE) in 10-year risk of deadly CVD [8–10]. The patients with high and very-high CVD risk are considered to be at risk for more severe clinical outcomes and higher recurrence rates [11–13], suggesting that we should pay sufficient attention to patients at high and very-high risk of CVD to prevent serious clinical outcomes.

It is shown that lipid-lowering medicines are benefit to ASCVD [14, 15]. Statin, an HMG-CoA reductase inhibitor, is the most effective therapeutic intervention to lower low-density lipoprotein cholesterol (LDL-C) [16]. However, patients with high CVD risk did not achieve sufficient statins treatment because of their side-effects [17]. The HPS2-THRIVE study showed that the incidence of adverse reactions was higher in Chinese population than in Europe and United States for the same dose of statin [18]. Furthermore, a doubling dose of statin was associated with greater lowering of LDL-C only by 4% to 6% and non-HDL-C by 3% to 6% [19]. The LDL-C levels of many high risk and very high-risk CVD patients still did not up to scratch after maximal tolerated statin therapy. Due to the existence of these conditions, intensive lipid-lowering therapy by nonstatin drugs or statins combined with nonstatin drugs may be an important option for patients with high CVD risk and very-high CVD risk.

**Figure 1:**
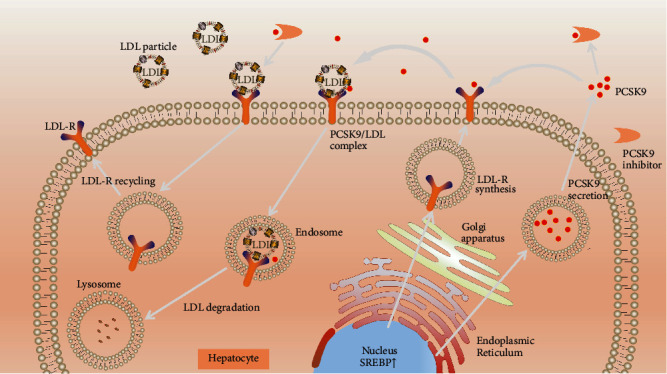
Mechanism of PCSK9 inhibitors: LDLR binds to circulating LDL particles then LDLR/LDL complexes are internalized in endosomes. In the endosome, LDL is carried to lysosome to be degraded when LDLR is recycled to the cell surface. When PCSK9 is present, it binds to LDLR to induce its internalization and degradation in lysosomes. This leads to decreased LDLR expression on the cell surface therefore increases circulating LDL-C levels.

PCSK9 is synthesized by hepatic inactive proprotein convertase and circulates either freely or bound to atherogenic lipoproteins, such as Lp (a) and LDL [20]. When PCSK9 binds directly to LDL receptor (LDLR), the LDL-C level in the circulation is elevated via degrading LDLR in the hepatocyte cell membrane [21] (Figure 1). Since the discovery of PCSK9, researchers gradually pay attention to inhibit the activity of this protein. The two monoclonal antibodies targeting PCSK9, alirocumab and evolocumab, have been approved in several countries around the world [20]. Data derived from the FOURIER trial on evolocumab and ODYSSEY OUTCOMES on alirocumab have indicated a clear clinical benefit in subjects at high CVD risk. Additionally, 2019 ESC/EAS guideline suggests that the treatments using PCSK9 inhibitors alone or in combination with statins clearly reduce the risk of recurrent ASCVD events in high-risk patients [8]. However, the benefit and safety of PCSK9 inhibitor administration on overall lipid levels of high and very-high cardiovascular risk patients is to be explored. Therefore, we performed a meta-analysis of randomized controlled trials (RCTs) to assess the clinical evidence for PCSK9 inhibitors.

## 2. Methods

This meta-analysis was conducted in accordance with the Preferred Reporting Items of Systematic Reviews and Meta-Analyses (PRISMA) statement (Supplementary Table S1) [22].

### 2.1. Research Strategy

Articles from PubMed/Medline, Embase databases, CENTRAL (Cochrane Central Register of Controlled Trials), and ClinicalTrials.gov were screened and selected up to June 2021. Additional searches were performed by scrutinizing relevant reviews and meta-analyses. The following keywords were used “PCSK9 inhibitor,” “cardiovascular disease,” “high risk,” and “lipid-lowering.” Trials were limited to RCTs and English literature (the search strategy of PubMed database is shown in Supplementary Table S2).

### 2.2. Literature Inclusion Criteria

#### 2.2.1. Research Design

RCTs were selected regardless of publication restrictions. Due to the different standards for high risk and very-high risk patients in the official guidelines each year, the inclusion and exclusion criteria for high risk and very-high risk patients in this study followed the guideline criteria for the year in which the trials were conducted.

#### 2.2.2. Research Subjects

The study included patients who had high and very-high risk of cardiovascular disease (P).

#### 2.2.3. Intervention

The control group received standard therapy with statins alone or ezetimibe alone or a combination therapy of statins and ezetimibe (C), while the experimental group received PCSK9 inhibitor besides the treatments applied in control group therapy (I).

#### 2.2.4. Outcome Indicators

Outcome indicators included all-cause mortality and cardiovascular mortality, percentage of decrease from baseline of serum LDL-C, total cholesterol (TC), triglyceride (TG), apolipoprotein B (ApoB), LP (a), non-HDL-C, high-density lipoprotein cholesterol (HDL-C), and apolipoprotein A1(ApoA1) (O).

### 2.3. Document Exclusion Criteria

The following were excluded: (1) duplicate articles, (2) case reports, (3) animal experimental studies, (4) review and observational studies, (5) trials with no relevant population, and (6) trials with no relevant outcome

### 2.4. Quality Assessment and Data Extraction

The included studies were subject to be appraised against the criteria for quality assessment recommended by the Cochrane systematic review [23], which consists of six domains: (1) random assignment method, (2) hidden assignment, (3) the use of blind method, (4) data completeness, (5) selective study results, and (6) biased presence. “Low or high risk” means a low or high risk of trial bias, and “unclear risk” means that sufficient information from the literature could not be obtained for risk of bias assessment. Quality assessment was performed by two independent investigators. Disagreements were solved by discussion or third-party judgement.

### 2.5. Statistical Analysis

Data were analyzed by Rev Man 5.1.0 software and Stata 16.0. Count data was assessed with risk ratio (RR) to determine the effect size, while mean difference (MD) was used to analyze the continuous data for determining the variance values and displaying the 95% confidence interval (CI_S_). The *P* value and I^2^ statistic of the heterogeneity test results shown in forest plot were used to ascertain whether the included studies were heterogeneous. If the heterogeneity was insignificant (*P* ≥ 0.1, *I*^2^ ≤ 50%), the fixed effects model was used to combine effect sizes; if the heterogeneity was significant (*P* < 0.1, *I*^2^>50%), effect sizes were combined using a random-effects model. We used sensitivity analysis to assess the stability of our results. Publication bias was examined visually and additional checks were performed by Begg's test.

### 2.6. Patient and Public Involvement Statement

This is a meta-analysis study with no patients involved.

## 3. Results

### 3.1. Literature Screening

At the beginning, 492 articles were included. Then, 51 duplicate articles were removed and another 338 articles were excluded by comparing their title and abstract against exclusion criteria. By then, 103 English articles were remained, whose full text were read and screened against exclusion and inclusion criteria. Finally, 14 trials with 52,586 patients were included in the meta-analysis. The process of literature screening was shown in Supplementary Figure S1.

### 3.2. Literature Quality Evaluation

A total of 52,586 experimental patients were included in this meta-analysis, with an average age of 61 years. Female patients accounted for 26.3%, and the average duration of treatment with PCSK9 inhibitor was 24 weeks. More general information and inclusion criteria of 14 trials were, respectively, shown in Table 1 and Supplementary Table S3, and the literature quality evaluations were presented in Supplementary Figures S2 and S3.

### 3.3. Meta-Analysis Outcome

#### 3.3.1. All-Cause Mortality

**Figure 2:**
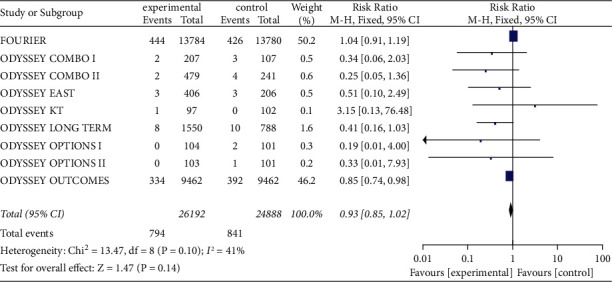
The all-cause mortality between two groups.

All of 14 studies (*n* = 51,080 patients) had demonstrated the clinical security of treatments with PCSK9 inhibitors. There was no significant heterogeneity (I^2^ = 41%, *P*=0.10) in the all-cause mortality of whole included patients, so meta-analysis was conducted using fixed effect model. The experimental group did not show significant change in all-cause mortality compared with that in control group [RR = 0.93, 95% CI (0.85, 1.02), *P*=0.14], as shown in Figure 2.

#### 3.3.2. Cardiovascular Mortality

**Figure 3:**
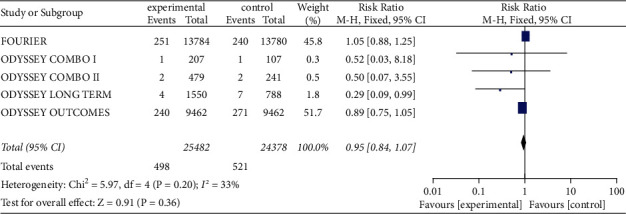
The cardiovascular mortality between two groups.

There was no significant heterogeneity (I^2^ = 33%, *P*=0.20) in the cardiovascular mortality of 49,860 patients, so meta-analysis was conducted using fixed effect model. The experimental group did not show significant change in cardiovascular mortality compared with that in control group [RR = 0.95, 95% CI (0.84, 1.07), *P*=0.36], as shown in Figure 3.

#### 3.3.3. LDL-C Level

**Figure 4:**
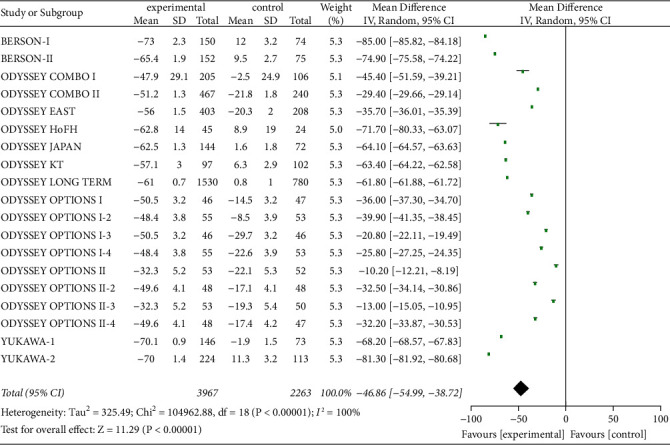
Comparison of the reduction of serum LDL-C (%) from baseline between control group and experimental group.

Nineteen studies include serum LDL-C levels as a parameter to examine PCSK9 inhibitor interventions. In total, 6,230 patients were included, and the heterogeneity was significant (*P* < 0.00001, I^2^ = 100%), so random-effects model was applied. Meta-analysis showed that the reduction of serum LDL-C level from baseline was significantly greater in experimental group of patients with high and very-high CVD risk than those in control group [*Z* = 11.29, MD = −46.86, 95% CI (−54.99 to −38.72), *P* < 0.00001], as shown in Figure 4.

#### 3.3.4. TC Level

**Figure 5:**
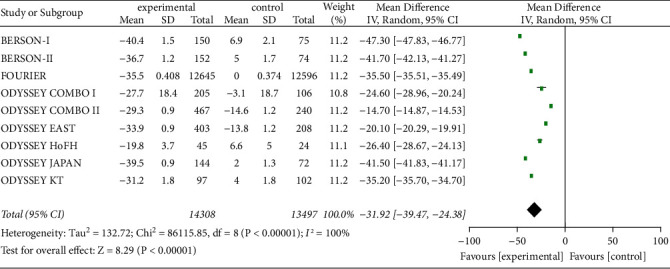
Comparison of the reduction of serum TC (%) from baseline between control group and experimental group.

As shown in Figure 5, nine studies with 27,805 patients were analyzed the effect of PCSK9 inhibitor on TC level, and the heterogeneity was significant (*P* < 0.00001, I^2^ = 100%). Data showed that the serum TC level from baseline in high and very-high CVD risk patients was reduced even more in experimental group than in control group [*Z* = 8.29, MD = −31.92, 95% CI (−39.47 to −24.38), *P* < 0.00001].

#### 3.3.5. TG and LP(a) Level

**Figure 6:**
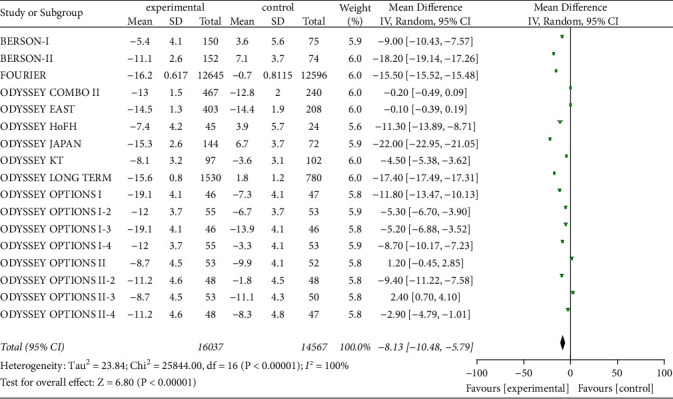
Comparison of the reduction of serum TG (%) from baseline between control group and experimental group.

**Figure 7:**
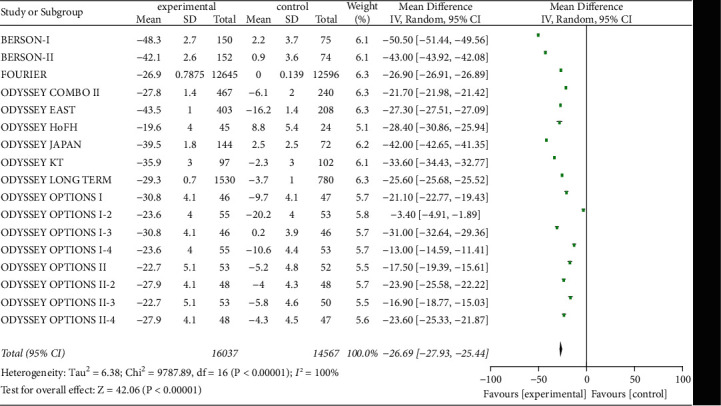
Comparison of the reduction of serum Lp (a) (%) from baseline between control group and experimental group.

Seventeen studies investigated serum TG and LP(a) levels in 30,604 patients to assess the effect of PCSK9 inhibitors on intervention for high- and very high-risk CVD. Heterogeneity was significant (*P* < 0.00001, I^2^ = 100%), so a random-effects model was used. The results showed that the reduction in serum TG and LP(a) levels from baseline were markedly greater in experimental group than in control group [*Z* = 6.80, MD = −8.13, 95% CI (−10.48 to −5.79), *P* < 0.00001] and [*Z* = 42.05, MD = −26.69, 95% CI (−27.93 to −25.44), *P* < 0.00001], as shown in Figures 6 and 7.

#### 3.3.6. Non-HDL-C and ApoB Levels

**Figure 8:**
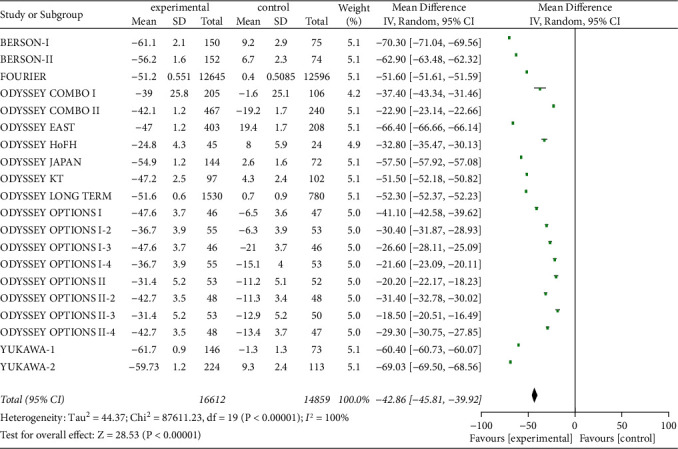
Comparison of the reduction of serum non-HDL-C (%) from baseline between control group and experimental group.

**Figure 9:**
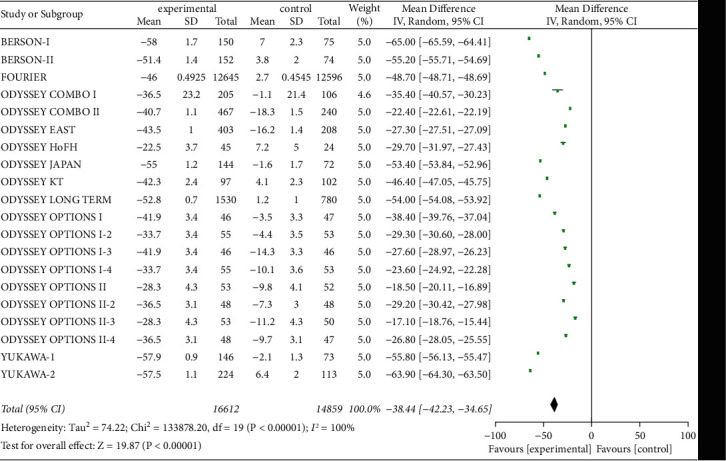
Comparison of the reduction of serum ApoB (%) from baseline between control group and experimental group.

Twenty studies on serum non-HDL-C and ApoB levels were included to evaluate the effect of PCSK9 inhibitors, as shown in Figures 8 and 9. A total of 31,471 patients were included, with significant heterogeneity (*P* < 0.00001, *I*^2^ = 100%), so a random-effects model was used. The results suggested that the decreases in serum non-HDL-C and ApoB levels from baseline were substantially greater in experimental group than in control group [*Z* = 28.53, MD = −42.86, 95% CI (−45.81 to −39.92), *P* < 0.00001] and [*Z* = 19.87, MD = −38.44, 95% CI (−42.23 to −34.65), *P* < 0.00001].

#### 3.3.7. HDL-C Level

**Figure 10:**
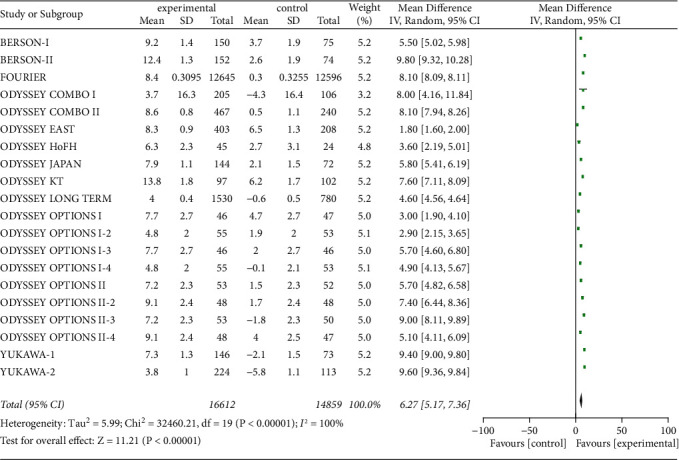
Comparison of the rise of serum HDL-C (%) from baseline between control group and experimental group.

Twenty studies included serum HDL-C level to assess the effect of PCSK9 inhibitor interventions. Overall, 31,471 patients were included, and the heterogeneity was significant (*P* < 0.00001, I^2^ = 100%), so random-effects model was used. Meta-analysis showed that the raise of serum HDL-C level from baseline was significantly greater in experimental group than those in control group [*Z* = 11.21, MD = 6.27, 95% CI (5.17 to 7.36), *P* < 0.00001], as shown in Figure 10.

#### 3.3.8. ApoA1 Level

**Figure 11:**
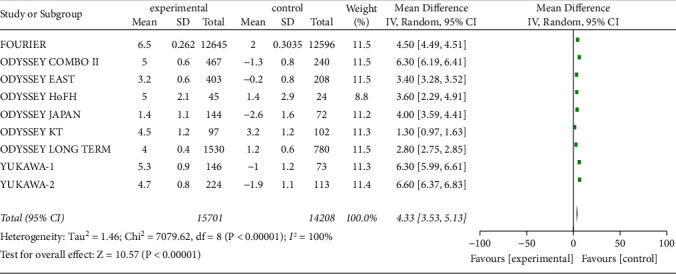
Comparison of the rise of serum ApoA1 (%) from baseline between control group and experimental group.

A total of nine studies (29,909 patients) reported serum ApoA1 level to assess the effect of PCSK9 inhibitors. Data heterogeneity was significant (*P* < 0.00001, I^2^ = 100%), so a random-effects model was used. Meta-analysis showed that the increase in serum ApoA1 level from baseline was considerably greater in high CVD patients with alirocumab or evolocumab [*Z* = 10.57, MD = 4.33, 95% CI (3.53 to 5.13), *P* < 0.00001], as shown in Figure 11.

#### 3.3.9. Publication Bias

Publication bias was showed in Supplementary Table S4 and Supplementary Figures S4 and S5. No publication bias was identified by visually inspected the funnel plot or by Begg's test in analyzing the outcomes of all-cause death, cardiovascular mortality, LDL-C, TC, TG, LP (a), non-HDL-C, ApoB, HDL-C, and ApoA1.

#### 3.3.10. Additional Analyses

In the sensitivity analysis, we addressed the influence of each trial, investigating whether omitting the studied trial would significantly alter the pooled results of the meta-analysis. When each trial was deleted one by one, the original overall estimates did not show any deviations, which meant that our findings were robust (Supplementary Table S5).

## 4. Discussion

### 4.1. Efficacy of PCSK9 Inhibitors

This study aimed to conduct a systematic review and meta-analysis for evaluating the effects of treatment with PCSK9 inhibitors on the blood levels of high and very-high CVD risk patients. The existing lipid-lowering drugs have a single target of intervention, and most of them only have a good modulation effect on one or several of them but cannot achieve the simultaneous modulation of multiple lipids. Our meta-analysis found that PCSK9 combination therapy resulted in beneficial and comprehensive modulation of serum LDL-C, TC, TG, ApoB, LP (a), non-HDL-C, HDL-C, and ApoA1 levels in patients with CVD high risk.

The results showed that the all-cause mortality and cardiovascular mortality were not statistically different between with or without PCSK9 inhibitors in high and very-high CVD risk patients. This might be due to the insufficient duration of clinical trials to know the effect of long-term PCSK9 inhibitor use on patient mortality. Interestingly, the addition of PCSK9 inhibitor reduced serum levels of LDL-C and TC compared with background lipid-lowering treatments without it. Accumulation of LDL and other ApoB members is known risk factors of ASCVD [37]. Recently, other meta-analysis showed that the addition of PCSK9 inhibitors to high-intensity statin therapy can significantly further reduce the expression of serum LDL-C in patients of very-high CVD risk, which is consistent to the same as our results [38, 39]. A meta-analysis study found that, for every 1 mmol/L reduction in LDL-C, the incidence of heart attacks and ischemic strokes was reduced by more than one-fifth, and there was no evidence of any threshold within the cholesterol range studied [40]. One study found that patients treated with high doses of cholesterol-lowering drugs were at increased risk of osteoporosis and cerebral hemorrhage [41, 42]. A prospective cohort study in Copenhagen that included 108,000 people confirmed a U-shaped association between LDL-C levels and all-cause mortality, with either high or low LDL-C associated with an increased risk of all-cause mortality [43]. Therefore, focusing only on LDL-C reduction in patients at high risk of CVD is one-sided, and we need to assess lipid levels comprehensively.

Despite the reductions of LDL-C with maximally tolerated statins, many people still experience dangerous cardiovascular events [44], which may be, in part, due to high serum TG level. Elevated serum levels of TGs or TG-rich lipoproteins and their remnants are all independently risk factors for CVD [45, 46]. The possible mechanisms involve the production of proinflammatory cytokines, excessive release of free fatty acids, impairment of coagulation factors, and fibrinolysis [47]. In this study, we considered that the addition of PCSK9 inhibitor distinctly decreased serum TG levels in high and very-high risk CVD patients.

Lp (a) is an LDL particle with covalently linked to apoB-100 [48]. Elevated concentration of Lp (a) is mainly caused by increased production of Lp (a) particles from liver and associates with the risk of CVD [49–51]. Lp (a) accumulation promotes foam cell formation, inflammation, oxidative stress, and thrombosis at the subendothelial space [51] and then leads to a series of serious adverse cardiovascular consequences like coronary artery disease and heart failure, despite optimal LDL-C management [50]. Although conventional drug treatments, such as statins, niacin, and cholesteryl ester transfer protein, failed to reduce serum Lp (a) levels back to normal range, PCSK9 inhibitors showed satisfactory results in lowering Lp (a) [52]. Our data demonstrated that treatment with alirocumab or evolocumab reduced serum LP (a) levels in high CVD risk patients. However, the mechanism of PCSK9 inhibitors on reducing LP (a) is still not clear but may be through adjusting LDLR activity [53].

Recently, the Italian Federation of Cardiology suggested that non-HDL-C is more closely related to CVD than LDL-C, which could be used as a prognostic marker for atherosclerosis treatment [54]. Non-HDL-C reflects the cholesterol content of atherogenic apoB-containing lipoproteins, which includes VLDL, intermediate density lipoprotein (IDL), LDL, chylomicron remnants, and LP (a) [55]. Non-HDL-C is of an advantage over LDL-C since it includes lipoprotein with remnant cholesterol and is free from triglyceride variability [5]. Additionally, ApoB, the major structural protein in LP (a), LDL, IDL, and VLDL, is used to quantify the total atherogenic lipoproteins. It was reported that non-HDL-C and apoB are superior to LDL-C in predicting ASCVD risk [56]. A study found that PCSK9 regulates apoB secretion by affecting autophagy, which might be a mechanism of PCSK9 inhibitors in reducing apoB [57]. Our meta-analysis discovered that the intervention of PCSK9 inhibitors notably further reduced non-HDL-C and ApoB levels in patients with high CVD risk.

ApoA1 is the most abundant protein component of HDL, and reduced plasma level of ApoA1 has been implicated as a CVD risk factor [58]. Raising serum HDL-C levels pharmacologically was included in lipid-lowering strategy to prevent the adverse CVD events since the discovery of HDL-C in the 1960s [59]. Thus, HDL-C is a consistent, robust, and independent marker to predict CVD risk by both the European and American Heart Association [9]. Epidemiological and genetic studies have suggested a positive correlation between PCSK9 and HDL-C levels, possibly through reduced uptake of ApoE-containing HDL particles [60]. In this study, we indicated that the PCSK9 inhibitors memorably increased the levels of HDL and ApoA1 in high CVD risk patients.

### 4.2. Limitations

① The size of samples in included studies varied greatly from 69 to 27564, which led to significant heterogeneity in the results. ② Some studies did not adequately report allocation concealment, which might lead to exaggeration of curative effect. ③ Our meta-analysis was based on trials rather than on individuals. Individuals' data allow us to perform a more rational subgroup analysis to explore sources of heterogeneity between treatment groups. ④ The dose and frequency of administration varies from study to study, and this may introduce a bias. ⑤ The patient's background medication used different types and doses of statins, which may also have an impact on the patient's final lipid levels. ⑥ Only a few RCTs reported changes in ApoA1 levels. Therefore, the results of this part of the data should be treated with caution.

### 4.3. Application Prospects

Two PCSK9 inhibitors were used in this study, including alirocumab and evolocumab. Alirocumab and evolocumab were generally well tolerated, except for adverse reactions such as flu-like symptoms, upper respiratory tract infections, and nasopharyngitis [61–63]. Therefore, PCSK9 may be a good choice for future lipid-lowering therapy. In addition, serum LP (a), TG, HDL-C, and other indicators are also related to cardiovascular events, which may also be used as alternative indicators to assess the risk and efficacy of CVD [62]. These benefits were confirmed by clinical studies, which reveals that PCSK9 inhibitors improve cardiovascular prognosis through multiple mechanisms [62]. In addition, recent studies have found that PCSK9 inhibitors may inhibit inflammation to some extent [61]. At present, PCSK9 inhibitor treatment combined with statins has received extensive attention, and a meta-analysis suggested that the addition of PCSK9 inhibitors to statins significantly promotes the regression of total atheroma volume [64]. To conclude, the future development of PCSK9 inhibitors is promising. To better understand the mechanism of PCSK9 in LDLR degradation, new molecular pathways to regulate its binding to LDLR, including PCSK9 small molecule stem RNA and PCSK9 vaccine, are to be explored. Such as inclisiran, an siRNA inhibitor of PCSK9, has the advantages of reduced LDL-C levels, good tolerability, and a low incidence of adverse events [65], which could provide new ideas for more effective PCSK9 inhibition.

## 5. Conclusion

This meta-analysis systematically describes the effect of addition of PCSK9 inhibitor to conventional lipid-lowering agents on regulating blood lipid levels in patients with high and very-high risk of CVD. The included results show that PCSK9 inhibitor decreased serum levels of LDL-C, TC, apoB, non-HDL-C, LP (a), TG, and increased levels of HDL-C and ApoA1 compared with conventional therapy alone.

## Figures and Tables

**Table 1:** Characteristics of the 14 trials identified in the literature search.

Included studies	Duration (weeks)	Sample size	Female	Mean age, years	Baseline LDL-C, mg/dl	Background therapy	Intervention (experimental group *vs* control group)	Participants
BERSON [24]	12	451	51.0	61	89.1 (34.9)	Atorvastatin 20 mg/d	Evolocumab (140 mg Q2 W or 420 mg monthly) + SOC *VS* SOC	Patients with T2DM and hyperlipidaemia or mixed dyslipidemia
FOURIER [25]	48	27564	24.6	63	92.0 (80–109)	Atorvastatin 20 mg daily, with or without ezetimibe	Evolocumab (140 mg Q2 W or 420 mg monthly) *VS* placebo	Patients with ASCVD and LDL-C>70 mg/dl or higher
ODYSSEY OUTCOMES [26]	48	18924	25.2	59	92.0 (31)	High-dose statin therapy or maximum tolerated statin	Alirocumab (75/150 mg Q2 W) *VS* placebo (Q2 W)	Patients with ACS, and LDL-C>70 mg/dl, non HDL-C> 100 mg/dl, or ApoB >80 mg/dl
ODYSSEY COMBO I [27]	24	316	32.7	63	94.8 (29.3)	Stable, maximally tolerated statin dose	Alirocumab (75/150 mg Q2 W) *VS* placebo (Q2 W)	Patients with high CV risk
ODYSSEY COMBO II [28]	52	720	27.15	62	108.2 (34.8)	Stable dose of statin	Alirocumab (75 mg Q2 W) *VS* ezetimibe (10 mg daily)	Patients with high CV risk
ODYSSEY HoFH [29]	12	69	49.6	43	295 (154.6)	Stable dose of statin	Alirocumab (150 mg Q2 W) *VS* placebo (Q2 W)	Patients with HoFH and LDL-C >70 mg/dl
ODYSSEY JAPAN [30]	24	216	38.2	61	142.8 (27.1)	Stable daily statin	Alirocumab (75 mg Q2 W) *VS* placebo	Patients with heFH, or non-FH at high CV risk
ODYSSEY KT [31]	24	199	17.5	61	97.0 (27.8)	Maximally tolerated statin	Alirocumab (75/150 mg Q2 W) *VS* placebo (Q2 W)	Patients with hypercho- lesterolemia at high CV risk
ODYSSEY LONG TERM [32]	24	2341	38.3	61	122.7 (42.6)	High-dose statin therapy or maximum tolerated statin	Alirocumab (150 mg Q2 W) *VS* placebo	Patients at high risk for CV events
ODYSSEY OPTIONS I [33]	12/24	310	35.25	64	109.5 (36.0)	Atorvastatin 20 or 40 mg	Alirocumab (75/150 mg Q2 W) + atorvastatin *VS* ATV alone/ezetimibe + ATV	Patients with hypercholesterolemia, very-high or high CVD risk
ODYSSEY OPTIONS II [34]	12/24	305	38.6	61	113.1 (29.4)	Rosuvastatin 10 or 20 mg	Alirocumab (75/150 mg Q2 W) + RSV *VS* RSV alone/ezetimibe + RSV	Patients with hypercholesterolemia, very-high or high CVD risk
ODYSSEY EAST [35]	24	615	25.0	59	110.8 (48.9)	Maximally tolerated statin therapy	Alirocumab (75/150 mg) *VS* ezetimibe (10 mg daily)	Patients with hypercholesterolemia, high CV risk
YUKAWA-1 [36]	12	219	43.5	61	138.7 (22.1)	Stable statin therapy	Evolocumab (420 mg monthly) + SOC *VS* SOC	Patients with hypercho-lesterolemic, high CV risk
YUKAWA-2 [36]	12	337	66.0	61	106.0 (32.1)	Stable statin therapy	Evolocumab (140 mg Q2 W or 420 mg monthly) + SOC *VS* SOC	Patients with hypercho-lesterolemic, high CV risk

CV: cardiovascular; ACS: acute coronary syndrome; HoFH : homozygous familial hypercholesterolemia; heFH: familial hypercholesterolemia; non-FH: nonfamilial hypercholesterolemia; T2DM: type 2 diabetes mellitus; SOC: standard of care; Q2 W: every 2 weeks

## Data Availability

The data used to support the findings of this study are included within the article.

## Supplementary Materials

Supplementary Materials Supplementary Table 1 (Table S1): PRISMA checklist. Supplementary Table 2 (Table S2): search strategy using PubMed database. Supplementary Table 3 (Table S3): inclusion criteria for patients. Supplementary Table 4 (Table S4): publication bias. Supplementary Table 5 (Table S5): sensitivity analysis. Supplementary Figure 1 (Figure S1): PRISMA flowchart of meta-analysis. Supplementary Figure 2 (Figure S2): individual bias assessment of included studies. Supplementary Figure 3 (Figure S3): summary bias assessment of included studies. Supplementary Figure 4 (Figure S4): funnel plot of the all-cause mortality. Supplementary Figure 5 (Figure S5): funnel plot of the cardiovascular mortality.
