# Supplementary material for: Effect of PCSK9 Inhibitor on Blood Lipid Levels in Patients with High and Very-High CVD Risk: A Systematic Review and Meta-Analysis
Source: Cardiol Res Pract. 2022 Apr 26;2022:8729003. doi: 10.1155/2022/8729003 (PMC9072011; doi:10.1155/2022/8729003)
Supplement: Supplementary Materials — Supplementary Table 1 (Table S1): PRISMA checklist. Supplementary Table 2 (Table S2): search strategy using PubMed database. Supplementary Table 3 (Table S3): inclusion criteria for patients. Supplementary Table 4 (Table S4): publication bias. Supplementary Table 5 (Table S5): sensitivity analysis. Supplementary Figure 1 (Figure S1): PRISMA flowchart of meta-analysis. Supplementary Figure 2 (Figure S2): individual bias assessment of included studies. Supplementary Figure 3 (Figure S3): summary bias assessment of included studies. Supplementary Figure 4 (Figure S4): funnel plot of the all-cause mortality. Supplementary Figure 5 (Figure S5): funnel plot of the cardiovascular mortality. [file 8729003.f1.zip › 8729003.f1/Supplement 1.docx]

**Supplementary 1**

**Table S1. PRISMA-P (Preferred Reporting Items for Systematic review and Meta-Analysis Protocols) 2015 checklist: recommended items to address in a systematic review protocol***

| Section and topic | Item No | Checklist item | Information reported | | Reported on page # |
| --- | --- | --- | --- | --- | --- |
|  |  |  | Yes | No |  |
| ADMINISTRATIVE INFORMATION | | |  |  |  |
| Title |  |  |  |  |  |
| Identification | 1a | Identify the report as a protocol of a systematic review | √ |  | 1 |
| Update | 1b | If the protocol is for an update of a previous systematic review, identify as such |  | √ | NA |
| Registration | 2 | If registered, provide the name of the registry (such as PROSPERO) and registration number |  | √ | NA |
| Authors |  |  |  |  |  |
| Contact | 3a | Provide name, institutional affiliation, e-mail address of all protocol authors; provide physical mailing address of corresponding author | √ |  | 1 |
| Contributions | 3b | Describe contributions of protocol authors and identify the guarantor of the review | √ |  | 13 |
| Amendments | 4 | If the protocol represents an amendment of a previously completed or published protocol, identify as such and list changes; otherwise, state plan for documenting important protocol amendments |  | √ | NA |
| Support |  |  |  |  |  |
| Sources | 5a | Indicate sources of financial or other support for the review | √ |  | 13 |
| Sponsor | 5b | Provide name for the review funder and/or sponsor | √ |  | 13 |
| Role of sponsor or funder | 5c | Describe roles of funder(s), sponsor(s), and/or institution(s), if any, in developing the protocol | √ |  | 13 |
| INTRODUCTION | | |  |  |  |
| Rationale | 6 | Describe the rationale for the review in the context of what is already known | √ |  | 3-4 |
| Objectives | 7 | Provide an explicit statement of the question(s) the review will address with reference to participants, interventions, comparators, and outcomes (PICO) | √ |  | 5 |
| METHODS | | |  |  |  |
| Eligibility criteria | 8 | Specify the study characteristics (such as PICO, study design, setting, time frame) and report characteristics (such as years considered, language, publication status) to be used as criteria for eligibility for the review | √ |  | 4-5 |
| Information sources | 9 | Describe all intended information sources (such as electronic databases, contact with study authors, trial registers or other grey literature sources) with planned dates of coverage | √ |  | 4 |
| Search strategy | 10 | Present draft of search strategy to be used for at least one electronic database, including planned limits, such that it could be repeated | √ |  | 4，Supplementary materials 1 |
| Study records |  |  |  |  |  |
| Data management | 11a | Describe the mechanism(s) that will be used to manage records and data throughout the review | √ |  | 4-5 |
| Selection process | 11b | State the process that will be used for selecting studies (such as two independent reviewers) through each phase of the review (that is, screening, eligibility and inclusion in meta-analysis) | √ |  | 4-5 |
| Data collection process | 11c | Describe planned method of extracting data from reports (such as piloting forms, done independently, in duplicate), any processes for obtaining and confirming data from investigators | √ |  | 4-5 |
| Data items | 12 | List and define all variables for which data will be sought (such as PICO items, funding sources), any pre-planned data assumptions and simplifications | √ |  | 5 |
| Outcomes and prioritize-ation | 13 | List and define all outcomes for which data will be sought, including prioritization of main and additional outcomes, with rationale | √ |  | 5 |
| Risk of bias in individual studies | 14 | Describe anticipated methods for assessing risk of bias of individual studies, including whether this will be done at the outcome or study level, or both; state how this information will be used in data synthesis |  | √ | NA |
| Data synthesis | 15a | Describe criteria under which study data will be quantitatively synthesised | √ |  | 6 |
|  | 15b | If data are appropriate for quantitative synthesis, describe planned summary measures, methods of handling data and methods of combining data from studies, including any planned exploration of consistency (such as I^2^, Kendall’s τ) | √ |  | 6 |
|  | 15c | Describe any proposed additional analyses (such as sensitivity or subgroup analyses, meta-regression) |  | √ | NA |
|  | 15d | If quantitative synthesis is not appropriate, describe the type of summary planned |  | √ | NA |
| Meta-bias(es) | 16 | Specify any planned assessment of meta-bias(es) (such as publication bias across studies, selective reporting within studies) | √ |  | Supplementary materials 2 |
| Confidence in cumulative evidence | 17 | Describe how the strength of the body of evidence will be assessed (such as GRADE) |  | √ | NA |

*** It is strongly recommended that this checklist be read in conjunction with the PRISMA-P Explanation and Elaboration (cite when available) for important clarification on the items. Amendments to a review protocol should be tracked and dated. The copyright for PRISMA-P (including checklist) is held by the PRISMA-P Group and is distributed under a Creative Commons Attribution Licence 4.0.**

**Table S****2.** Search strategy using Pubmed database

| **Search number** | **Query** |
| --- | --- |
| # 1 | PCSK9 inhibitor [MeSH Terms] |
| # 2 | Ralpancizumab [MeSH Terms] |
| # 3 | PCSK-9 inhibitors [Title/Abstract] |
| # 4  # 5 | proprotein convertase subtilisin-kexin type 9 inhibitors [Title/Abstract] |
| # 6 | RN317 compound [Title/Abstract] |
| # 7 | AMG145 [Title/Abstract] |
| # 8 | SAR236553 [Title/Abstract] |
| # 9 | lipemia [Title/Abstract] |
| # 10 | alirocumab [Title/Abstract] |
| # 11 | bococizumab [Title/Abstract] |
| # 12 | evolocumab [Title/Abstract] |
| # 13 | #1 OR #2 OR #3 OR #4 OR #5 OR #6 OR #7 OR #8 OR #9 OR #10 OR #11 OR #12 |
| # 14 | high risk [MeSH Terms] |
| # 15 | very high risk [MeSH Terms] |
| # 16 | # 14 OR # 15 |
| # 19  # 20 | cardiovascular diseases [MeSH Terms]  CVD [Title/Abstract] |
| # 21 | cardiovascular Disease [Title/Abstract] |
| # 22 | disease, Cardiovascular [Title/Abstract] |
| # 23 | diseases, Cardiovascular [Title/Abstract] |
| # 24 | #19 OR #20 OR #21 OR #22 OR #23 |
| # 25 | lipid-lowering treatment [Title/Abstract] |
| # 26 | lipid reduction treatment [Title/Abstract] |
| # 27 | lipid reduction [Title/Abstract] |
| # 28 | lipid-lowering [Title/Abstract] |
| # 29 | #25 OR #26 OR #27 OR #28 |
| # 30 | #13 AND #16 AND # 24 AND # 29 |

**Table S3**. Inclusion criteria for patients

| Included Studies | Reasons for patient inclusion |
| --- | --- |
| BERSON [25] | patients between 18 and 80 years old with T2DM, meet any of the following conditions:(a) HbAIc ≤ 10% and TG ≤ 400 mg/Dl. (b) patients treated with statins LDL-C ≥100 mg/dL. (c) patients treated without statins LDL-C ≥130 mg/dL. |
| FOURIER [26] | patients between 40 and 85 years old with evident ASCVD (history of MI, nonhemorrhagic stroke, or symptomatic peripheral artery disease, or other characteristics that placed them at higher cardiovascular risk). |
| ODYSSEY OUTCOMES [27] | patients aged ≥40 years old, with recent ACS (≤12 months) meanwhile meet any of the following conditions: (a) LDL-C>70 mg/dl. (b)Non HDL-C> 100 mg/dl. (c) ApoB > 80 mg/dl. |
| ODYSSEY COMBO I [28] | patients aged ≥18 years, meet any of the following conditions: (a) serum LDL-C ≥70 mg/dL and established CVD. (b) serum LDL-C ≥100 mg/dL with CHD risk equivalents. |
| ODYSSEY COMBO II [29] | meet any of the following conditions: (a) patients with CVD and serum LDL-C ≥70 mg/dL. (b) patients were at high cardiovascular risk and had serum LDL-C≥100 mg/dL. |
| ODYSSEY HoFH [30] | patients with HoFH and serum LDL-C ≥70 mg/dL. |
| ODYSSEY JAPAN [31] | meet any of the following conditions: (a) adults with heFH with or without a history of documented CAD. (b) patients with non-FH at high CV risk with a history of documented CAD, or classified as JAS category III[42] (primary prevention). |
| ODYSSEY KT [32] | patients with history of CVD, moderate CKD, or diabetes with multiple risk factors. |
| ODYSSEY LONG TERM [33] | Patients ≥18 years old with serum LDL-C ≥70 mg /dL and meet any of the following conditions: (a) with HoFH. (b) with established CHD. |
| ODYSSEY OPTIONS I [34] | patients ≥18 years old and meet any of the following conditions: (a) very-high risk of CVD (CHD, or T2DM with target organ damage) and serum LDL-C ≥ 70 mg/dL. (b) high risk (with other risk factors, moderate CDK, or diabetes with no target organ damage ^a^) and serum LDL-C ≥ 100mg/dL. |
| ODYSSEY OPTIONS II [35] | patients ≥18 years old and meet any of the following conditions: (a) very-high risk of CVD (CHD, or T2DM with target organ damage) and serum LDL-C ≥ 70 mg/dL. (b) high risk (with other risk factors, moderate CKD, or diabetes with no target organ damage ^a^) and serum LDL-C ≥ 100mg/dL. |
| ODYSSEY EAST [36] | meet any of the following conditions: (a) patients with a history of CVD and LDL-C levels were ≥70 mg/dL. (b) without a history of CVD but baseline serum LDL-C ≥100 mg/dL. |
| YUKAWA-1 [37] | patients ≥20 years old, and serum TGs ≤400mg/Dl, LDL-C ≥115mg/dL. |
| YUKAWA-2 [37] | patients ≥20 years old, and serum TGs ≤400mg/Dl, LDL-C ≥100mg/dL. |
| MI: myocardial infarction; HoFH: Homozygous familial hypercholesterolemia; CHD: coronary heart disease; CKD: chronic kidney disease; heFH: familial hypercholesterolemia; CKD: chronic kidney disease; T2DM: type 2 diabetes; Other risk factors: including smoking, hypertension or dyslipidemia, etc; ^a^Target organ damage: microalbuminuria, retinopathy, or neuropathy. | |

Table S4. Publication bias

|  | **Begg's tests (P value)** |
| --- | --- |
| **All-cause death** | 0.2515 |
| **Cardiovascular mortality** | 0.8065 |
| **LDL-C** | 1.9999 |
| **TC** | 1.7485 |
| **TG** | 1.9994 |
| **Lp (a)** | 1.9966 |
| **Non HDL-C** | 1.9996 |
| **ApoB** | 1.9987 |
| **HDL-C** | 0.0644 |
| **ApoA1** | 0.6022 |

Table S5. Sensitivity analyse

|  | RISK RATIO [95% CI] | | | | | | | | | |
| --- | --- | --- | --- | --- | --- | --- | --- | --- | --- | --- |
| Study excluded | **All-cause death** | **Cardiova-scular mortality** | **LDL-C** | **TC** | **TG** | **Lp (a)** | **Non HDL-C** | **ApoB** | **HDL-C** | **ApoA1** |
| BERSON-Ⅰ | — | — | -44.73  [-53.04,-36.41] | -29.99  [-38.08,-21.90] | -8.08  [-10.50, -5.66] | -25.21  [-26.33, -24.08] | -41.40  [-44.39,-38.41] | -37.03  [-40.90,-33.16] | 6.31  [5.18,7.43] | — |
| BERSON-Ⅱ | — | — | -45.29  [-53.73,-36.85] | -30.69  [-38.92,-22.46] | -7.49[-9.92, -5.07] | -25.65  [-26.87,-24.44] | -41.79  [-44.81,-38.78] | -37.55  [-41.46,-33.63] | 6.07  [4.95,7.20] | — |
| FOURIER | 0.82 [0.71,0.94] | 0.86  [0.73,1.02] | — | -31.47  [-40.51,-22.42] | -7.65[-13.04, -2.27] | -26.46  [-29.24,-23.69] | -42.27  [-48.85,-35.69] | -37.86  [-45.35,-30.37] | 6.17  [5.00,7.33] | 4.30  [2.95.5.65] |
| ODYSSEY COMBO I | 0.93  [0.85,1.03] | 0.95  [0.84,1.07] | -46.94  [-55.29,-38.58] | -32.81  [-40.80,-24.82] | — | — | -43.10  [-46.11,-40.10] | -38.58  [-42.47,-34.70] | 6.21  [5.10,7.32] | — |
| ODYSSEY COMBO II | 0.94  [0.85,1.03] | 0.95  [0.84,1.07] | -47.81  [-54.77,-40.86] | -34.12  [-40.26.-27.98] | -8.66[-10.59, -6.72] | -27.04  [-28.28,-25.81] | -44.18  [-46.12,-42.24] | -39.32  [-42.40,-36.25] | 6.17  [5.01,7.32] | 4.08  [3.23,4.92] |
| ODYSSEY EAST | 0.93 [0.85,1.03] | — | -47.48  [-55.78,-39.18] | -33.41  [-41.64,-25.18] | -8.66[-10.59, -6.74] | -26.62  [-27.97,-25.27] | -41.62  [-44.50,-38.74] | -39.04  [-42.49,-35.60] | 6.51  [5.43,7.59] | 4.45  [3.57,5.33] |
| ODYSSEY HoFH | — | — | -45.55  [-53.90,-37.20] | -32.61  [-40.62,-24.60] | -7.94[-10.36, -5.53] | -26.60  [-27.87,-25.32] | -43.38  [-46.40,-40.36] | -38.89  [-42.78,-35.01] | 6.40  [5.28,7.52] | 4.40  [3.56,5.24] |
| ODYSSEY JAPAN | — | — | -45.90  [-54.62,-37.18] | -30.72  [-39.11,-22.32] | -7.25[-9.67, -4.84] | -25.72  [-26.88,-24.57] | -42.08  [-45.12,-39.04] | -37.64  [-41.57,-33.72] | 6.29  [5.16,7.42] | 4.37  [3.52,5.23] |
| ODYSSEY KT | 0.93 [0.85,1.02] | — | -45.93  [-54.41,-37.46] | -35.51  [-39.71,-23.31] | -8.36  [-10.76, -5.96] | -26.24  [-27.51, -24.97] | -42.40  [-45.44,-39.37] | -38.01  [-41.92,-34.11] | 6.19  [5.07,7.32] | 4.72  [3.88,5.56] |
| ODYSSEY LONG TERM | 0.94 [0.85,1.03] | 0.96  [0.85,1.08] | -46.04  [-56.28,-35.81] | — | -7.53  [-12.46, -2.61] | -26.56  [-29.10,-24.02] | -42.24  [-48.37,-36.12] | -37.58  [-43.87,-31.29] | 6.36  [5.30,7.42] | 4.53  [3.68,5.38] |
| ODYSSEY OPTIONS I | 0.93 [0.85,1.03] | — | -47.46  [-55.82,-39.10] | — | -7.90  [-10.32, -5.49] | -27.02  [-28.30,-25.74] | -42.96  [-45.98,-39.94] | -38.44  [-42.33,-34.55] | 6.44  [5.31,7.56] | — |
| ODYSSEY OPTIONS I-2 | 0.93 [0.85,1.03] | — | -47.25  [-55.62,-38.87] | — | -8.31  [-10.72, -5.90] | -28.14  [-29.36,-26.91] | -43.52  [-46.54,-40.51] | -38.92  [-42.80,-35.04] | 6.45  [5.32,7.57] | — |
| ODYSSEY OPTIONS I-3 | 0.93 [0.85,1.03] | — | -48.31  [-56.59,-40.03] | — | -8.31  [-10.73.-5.90] | -26.43  [-27.71,-25.15] | -43.73  [-46.73,-40.72] | -39.01  [-42.89,-35.13] | 6.29  [5.17,7.42] | — |
| ODYSSEY OPTIONS I-4 | 0.93 [0.85,1.03] | — | -48.03  [-56.35,-39.71] | — | -8.10  [-10.51, -5.68] | -27.52  [-28.79,-26.26] | -43.99  [-46.99,-40.99] | -39.22  [-43.09,-35.35] | 6.34  [5.21,7.46] | — |
| ODYSSEY OPTIONS II | 0.93 [0.85,1.03] | — | -48.90  [-57.19,-40.61] | — | -8.71  [-11.11, -6.31] | -27.22  [-28.50,-25.95] | -44.05  [-47.06,-41.05] | -39.49  [-43.36,-35.61] | 6.30  [5.17,7.42] | — |
| ODYSSEY OPTIONS II-2 | 0.93 [0.85,1.03] | — | -47.66  [-56.01,-39.31] | — | -8.05  [-10.47, -5.64] | -26.85  [-28.13,-25.57] | -43.47  [-46.48,-40.46] | -38.92  [-42.81,-35.04] | 6.21  [5.08,7.33] | — |
| ODYSSEY OPTIONS II-3 | 0.93 [0.85,1.03] | — | -48.74  [-57.04,-40.44] | — | -8.78  [-11.18, -6.38] | -27.26  [-28.53,-25.99] | -44.14  [-47.14,-41.14] | -39.56  [-43.43,-35.69] | 6.12  [5.00,7.25] | — |
| ODYSSEY OPTIONS II-4 | 0.93 [0.85,1.03] | — | -47.67  [-56.02,-39.32] | — | -8.45  [-10.86, -6.04] | -26.87  [-28.15,-25.59] | -43.58  [-46.59.-40.57] | -39.05  [-42.93,-35.17] | 6.33  [5.20,7.45] | — |
| ODYSSEY OUTCOMES | 1.00 [0.88,1.13] | 1.01  [0.85,1.20] | — | — | — | — | — | — | — | — |
| YUKAWA-1 | — | — | -45.67  [-54.54,-36.80] | — | — | — | -41.92  [-44.95,-38.90] | -37.51  [-41.45,-33.58] | 6.09  [4.97,7.22] | 4.08  [3.23,4.93] |
| YUKAWA-2 | — | — | -44.93  [-53.29,-36.58] | — | — | — | -41.47  [-44.43,-38.51] | -37.09  [-40.95,-33.23] | 6.08  [4.95,7.22] | 4.04  [3.19,4.89] |
