# Supplementary material for: Effect of PCSK9 Inhibitor on Blood Lipid Levels in Patients with High and Very-High CVD Risk: A Systematic Review and Meta-Analysis
Source: Cardiol Res Pract. 2022 Apr 26;2022:8729003. doi: 10.1155/2022/8729003 (PMC9072011; doi:10.1155/2022/8729003)
Supplement: Supplementary Materials — Supplementary Table 1 (Table S1): PRISMA checklist. Supplementary Table 2 (Table S2): search strategy using PubMed database. Supplementary Table 3 (Table S3): inclusion criteria for patients. Supplementary Table 4 (Table S4): publication bias. Supplementary Table 5 (Table S5): sensitivity analysis. Supplementary Figure 1 (Figure S1): PRISMA flowchart of meta-analysis. Supplementary Figure 2 (Figure S2): individual bias assessment of included studies. Supplementary Figure 3 (Figure S3): summary bias assessment of included studies. Supplementary Figure 4 (Figure S4): funnel plot of the all-cause mortality. Supplementary Figure 5 (Figure S5): funnel plot of the cardiovascular mortality. [file 8729003.f1.zip › 8729003.f1/Supplement 2.docx]

**Supplementary2**





**Figure S1.** PRISMA flow chart of meta-analysis.





**Figure S2.** Individual bias assessment of included studies.


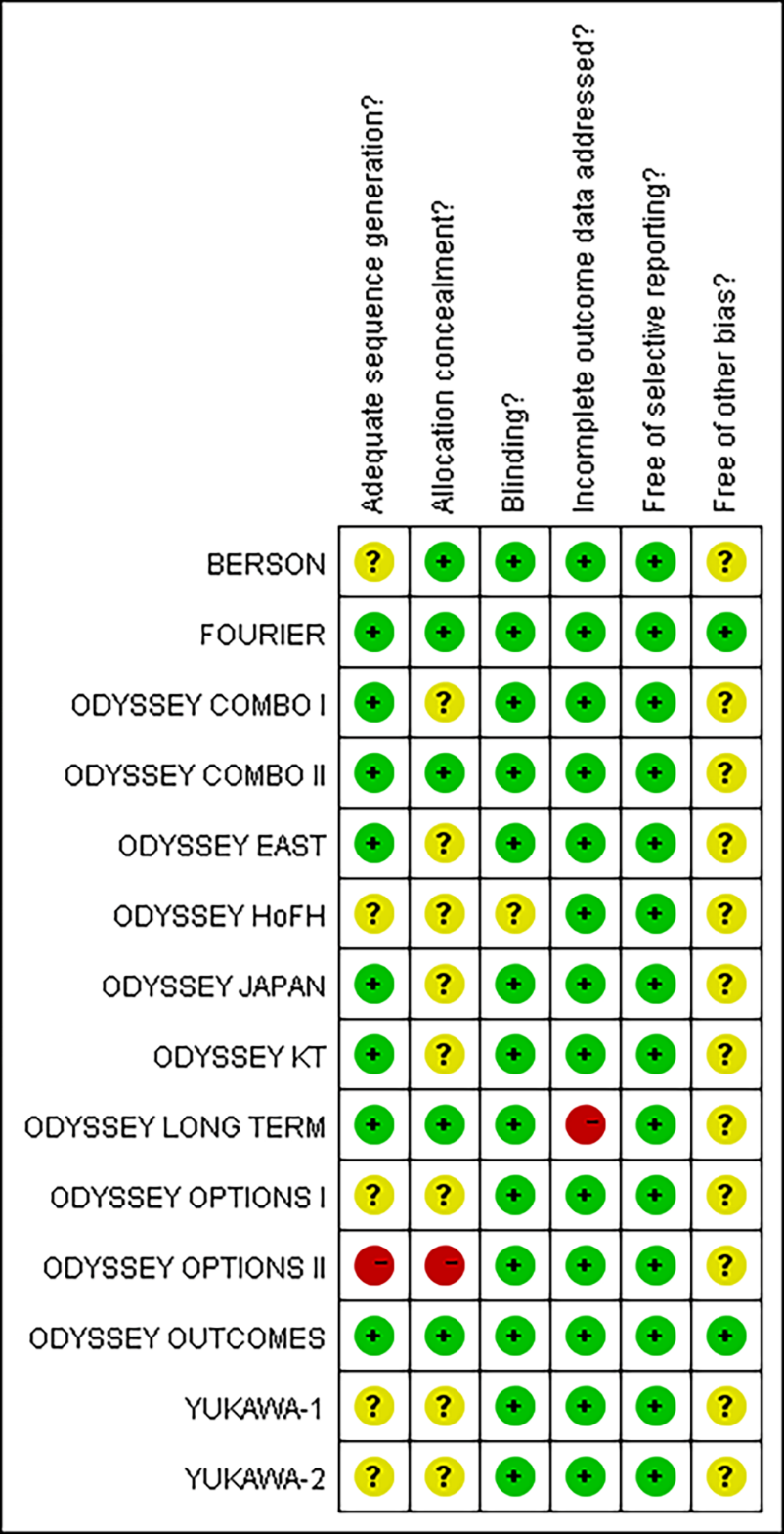


**Figure S3.** Summary bias assessment of included studies.





**Figure S4.**  Funnel plot of the all-cause mortality



 **Figure S5.**  Funnel plot of the cardiovascular mortality
